# A Review of Medical Student First-Author Publications in Plastic Surgery

**DOI:** 10.7759/cureus.43025

**Published:** 2023-08-06

**Authors:** Abhinav Lamba, Matthew D Rich, Joseph D Quick, Thomas J Sorenson, Ruth J Barta, Warren Schubert

**Affiliations:** 1 Department of Surgery, University of Minnesota Medical School, Minneapolis, USA; 2 Department of Plastic Surgery, New York University (NYU) Grossman School of Medicine, New York, USA; 3 Department of Craniofacial and Plastic Surgery, Gillette Children’s, Saint Paul, USA; 4 Department of Plastic and Hand Surgery, Regions Hospital, Saint Paul, USA

**Keywords:** first author publication, research productivity, residency match, medical education, plastic surgery

## Abstract

The integrated plastic surgery residency match is a highly competitive process. If performed wisely, medical research is an opportunity to differentiate applications from peers, and productivity is closely evaluated by residency programs. In this study, the authors aimed to characterize medical student research productivity for integrated plastic surgery residency programs and their respective medical schools. To this end, the authors performed a retrospective review of senior author publications from the 81 integrated plastic surgery programs from January 1, 2016, to December 31, 2020. The primary outcome was a publication with a medical student as the first author. Secondary outcomes included the number of faculty from each program, the geographic region of the program, and the ranking of associated medical schools. It was found that the average number of medical student first-author publications and faculty members per institution were 14.0 and 11.0, respectively. There was a positive correlation between the number of faculty members and several medical student first-author publications for a program (*R* = 0.54, *P* < 0.0001). The average number of medical student first-author publications was higher in the top 25 programs than for the remaining programs (*P* < 0.001), and most medical student first-author publications in the United States were produced by 10 plastic surgery programs. From these findings, it was concluded that these programs associated with higher-ranking medical schools produce greater numbers of medical student first-author publications. These analyses of medical student academic productivity should be a highly useful guide for current and future medical students as they strategize their successful match into plastic surgery.

## Introduction and background

Integrated plastic surgery is among the most competitive residencies available to current medical students due to limited residency spots and high medical student interest. As competition grows, opportunities for residency applicants to stand out to potential future program directors have become increasingly important. Though objective scores on the United States Medical Licensing Exam (USMLE) Step 1 exam and subjective *glowing* letters of recommendation are commonly cited, pre-residency research productivity, as measured by peer-reviewed publications, is also ranked very favorably by program directors [1-3]. Thus, medical students interested in pursuing a career in plastic surgery must accumulate publications before and/or during medical school to be strong candidates during the matching process. Department research volume and medical student involvement vary greatly by the department and medical school; therefore, two students with similar aspirations can have vastly different trajectories due to the largely uncontrollable circumstances surrounding them.

Knowing which institutions enable medical students to participate in meaningful research and produce first-author publications can help medical students pursue the best option when considering a mid-medical school *research year* and help premedical students pursue the medical school that most fit their career interests if they are lucky enough to have a choice. Thus, we aimed to provide objective data on the topic of medical student academic productivity in plastic surgery by quantifying the number of medical student first-author publications that have been published during five years from 2016 to 2020. We also further compared this number of publications for each program/department by department size, geographical region, and medical school ranking.

Information in this article was previously presented as a meeting abstract at the 67th annual meeting of the Plastic Surgery Research Council in Toronto, Ontario, Canada, from June 8 to 12, 2022.

## Review

Primary outcome

We performed a systematic literature review of medical student first-author publications from January 1, 2016, to December 31, 2020. First, we composed a list of plastic surgery programs using the Accreditation Council for Graduate Medical Education (ACGME). We considered all *integrated* plastic surgery programs with ACGME accreditation. To determine the list of faculty members for each program, we analyzed individual residency program websites. To be included, plastic surgery faculty members were required to have a doctorate degree (MD/DO/Ph.D.). We then conducted a literature search on each faculty member using PubMed Central and Scopus to obtain publication data. The search function was queried for each faculty member to identify senior author publications that were published during the study period. We then individually analyzed each faculty member’s senior author publications to determine the academic status of the first author.

The primary outcome of this study was medical student first-author publications. This was defined as a publication with the first author not having a medical degree (MD/DO). We determined this by reviewing the author's degrees after their name as listed on the publication. If the publication did not list the degree status for any author, additional publications were searched from the same approximate date to identify the first author’s degree status at the time of publication. In the situation of the first author with a higher degree that was not a medical degree (Ph.D., RN, or PA-C), these individuals were further evaluated to determine if they were part of a medical school program. This was done with various other social media and websites, including Twitter, LinkedIn, ResearchGate, and Google Scholar. If the author was determined to be part of a medical school program, they were included in the primary outcome. If not, they were excluded.

There were 81 integrated plastic surgery programs with ACGME accreditation in the United States and a total of 1,132 medical student first-author publications during the study period. Nationally, the average (±standard deviation [SD]) number of medical student first-author publications per program/department was 14.0 (±18.78). The University of Pennsylvania, Baylor College of Medicine, and New York University were the top three programs with 81, 72, and 70 publications, respectively. Baylor College of Medicine and Rutgers University were tied with the highest relative ratio of medical student first-author publications per faculty of 6.5:1. Seven programs averaged over 10 medical student first-author publications per year during the study period. Nine programs did not produce any medical student first-author publications during the study period (Table 1). 

**Table 1:** Ranking of 81 ACGME-certified plastic surgery residency programs based on the total medical student first-author publications from January 1, 2016, to December 31, 2020. ACGME, Accreditation Council for Graduate Medical Education

Program Name	Total medical student first-author publications	Number of faculty	Relative ratio
University of Pennsylvania Health System	81	16	5.1
Baylor College of Medicine	72	11	6.5
NYU Grossman School of Medicine	70	37	1.9
University of Michigan Health System	62	20	3.1
Yale-New Haven Medical Center	58	11	5.3
Brigham and Women's Hospital/Harvard Medical School	53	34	1.6
Stanford Health Care-sponsored Stanford University	53	16	3.3
Beth Israel Deaconess Medical Center	49	12	4.1
University of Southern California/LAC+USC Medical Center	41	8	5.1
Duke University Hospital	39	14	2.8
MedStar Health/Georgetown University Hospital	37	14	2.6
Washington University/Barnes-Jewish Hospital/St Louis Children's Hospital Consortium	31	15	2.1
New York Presbyterian Hospital (Columbia and Cornell Campus)	28	22	1.3
Rutgers Health/New Jersey Medical School	26	4	6.5
Johns Hopkins University/University of Maryland	24	20	1.2
Vanderbilt University Medical Center	21	13	1.6
Brown University	20	12	1.7
University of California, Los Angeles	18	16	1.1
University of Miami/Jackson Health System	17	12	1.4
McGaw Medical Center of Northwestern University	15	22	0.7
University of Pittsburgh Medical Center Medical Education	15	20	0.8
Rush University Medical Center	14	7	2.0
University of Chicago	14	11	1.3
Montefiore Medical Center/Albert Einstein College of Medicine of Yeshiva University	13	7	1.9
Spectrum Health/Michigan State University	13	24	0.5
Cleveland Clinic Foundation	12	22	0.5
Mayo Clinic College of Medicine and Science (Rochester)	11	12	0.9
Medical University of South Carolina College of Medicine	11	6	1.8
University of Texas Southwestern Medical Center	11	17	0.6
University of Wisconsin Hospitals and Clinics	11	12	0.9
Zucker School of Medicine at Hofstra/Northwell	10	9	1.1
Indiana University School of Medicine	9	14	0.6
University of Colorado School of Medicine	9	12	0.8
Case Western Reserve University/University Hospitals Cleveland Medical Center	8	15	0.5
Louisiana State University School of Medicine	8	10	0.8
University of Florida	8	7	1.1
University of Rochester	8	7	1.1
Ohio State University Hospital	7	17	0.4
Oregon Health & Science University	7	10	0.7
Penn State Milton S Hershey Medical Center	7	9	0.8
University of California, San Francisco	7	14	0.5
University of California, Irvine	6	8	0.8
University of Cincinnati Medical Center/College of Medicine	6	7	0.9
University of Kansas School of Medicine	6	12	0.5
University of Washington	6	17	0.4
Lahey Clinic	5	7	0.7
Lehigh Valley Health Network/University of South Florida College of Medicine	5	9	0.6
Mayo Clinic College of Medicine and Science (Arizona)	5	8	0.6
University of California, Davis	5	6	0.8
University of Virginia Medical Center	5	7	0.7
Emory University School of Medicine	4	8	0.5
Nassau University Medical Center/Stony Brook University	4	8	0.5
St Louis University School of Medicine	4	5	0.8
University of Kentucky College of Medicine	4	5	0.8
University of Utah Health	4	8	0.5
Prisma Health/University of South Carolina School of Medicine, Columbia	3	11	0.3
University of Nevada Las Vegas School of Medicine	3	5	0.6
University of New Mexico School of Medicine	3	1	3.0
University of North Carolina Hospitals	3	6	0.5
University of Texas Medical Branch Hospitals	3	9	0.3
Virginia Commonwealth University Health System	3	9	0.3
Wake Forest University School of Medicine	3	10	0.3
Albany Medical Center	2	7	0.3
Dartmouth- Hitchcock/Mary Hitchcock Memorial Hospital	2	7	0.3
Medical College of Wisconsin Affiliated Hospitals	2	16	0.1
University of Tennessee College of Medicine	2	8	0.3
Carilion Clinic- Virginia Tech Carilion School of Medicine	1	5	0.2
Icahn School of Medicine at Mount Sinai	1	9	0.1
Loma Linda University Health Education Consortium	1	21	0.0
Southern Illinois University	1	5	0.2
University of Massachusetts	1	8	0.1
University of Missouri, Columbia	1	4	0.3
Cooper Hospital-University Medical Center	0	8	0.0
Geisinger Health System	0	7	0.0
Loyola University Medical Center	0	4	0.0
Texas A&M College of Medicine-Scott and White Medical Center, Temple	0	6	0.0
University of California, San Diego	0	6	0.0
University of Minnesota	0	4	0.0
University of Mississippi School of Medicine	0	7	0.0
West Virginia University School of Medicine	0	4	0.0
Wright State University	0	3	0.0

Secondary outcomes

Programs were also assembled into the following stratification groups and statistically analyzed. Department size was determined by the number of plastic surgery faculty in the department. Faculty members who were not board eligible or board certified in plastic surgery, emeritus faculty, adjunct faculty, and exclusively research faculty were excluded from the study. The plastic surgery program region was determined according to prior studies [4-6]. Finally, the ranking of each program and its associated medical school was determined through the U.S. News & World Report (USNWR) and the National Institutes of Health (NIH) Blue Ridge Institute for Medical Research.

Department Size

**Figure 1:**
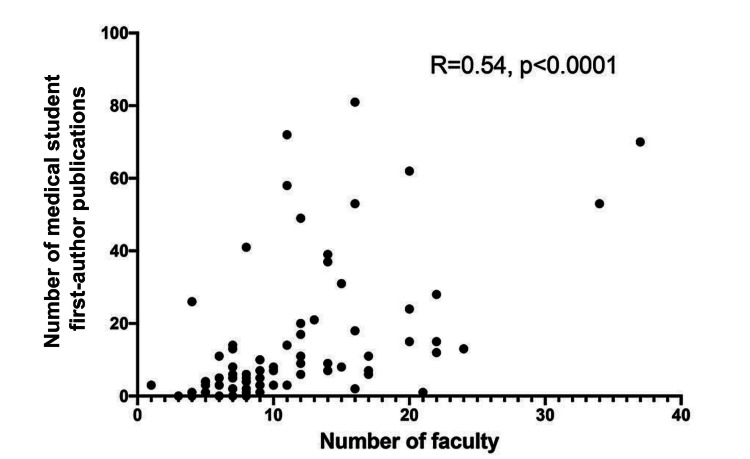
Medical student first-author publication volume is associated with the number of plastic surgery department faculty members. Scatterplot showing the correlation between the number of medical student first-author plastic surgery publications from January 1, 2016, to December 31, 2020, and the number of faculty members in the plastic surgery department of an ACGME-certified plastic surgery residency program (*n* = 81). Pearson correlation coefficient (*R*) and level of significance (*P*-value) are shown. ACGME, Accreditation Council for Graduate Medical Education

Nationally, the average (±SD) department size was 11.0 (±6.5) faculty members. The three largest departments were New York University, Harvard University, and Michigan State University with 37, 34, and 24 faculty members, respectively. There was a statistically significant positive correlation between the number of medical student first-author publications and the number of departmental faculty (*R* = 0.54, *P* < 0.0001), as indicated by Pearson’s coefficient (Figure 1).

Geographic Region

**Figure 2:**
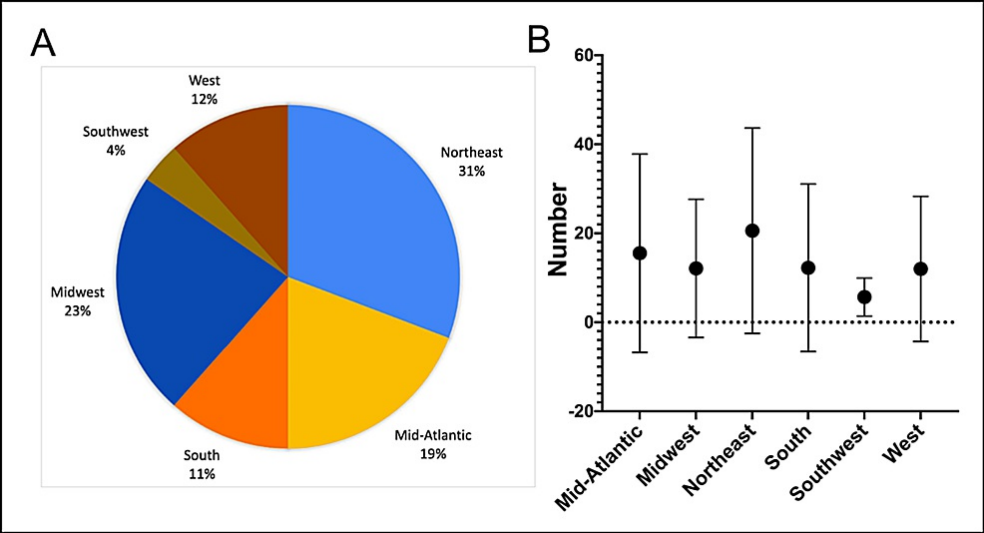
Geographic variation in the medical student first-author publication volume. (A) Pie chart depicting the percentage of the top 25 plastic surgery programs located in different regions. (B) Scatterplot representing the number of medical student first-author publications from January 1, 2016, to December 31, 2020, by plastic surgery department region. There was no significance in comparing these regions with a ANOVA. ANOVA, one-way analysis of variance

We also divided programs according to their geographic location. The number of programs by region was as follows: Mid-Atlantic (*n *= 13), Midwest (*n *= 16), Northeast (*n *= 17), South (*n *= 16), Southwest (*n *= 6), and West (*n *= 13). The region with the highest average (± SD) medical student first-author publications was the Northeast (20.6 ± 23.0), and the region with the highest average (± SD) faculty was the Mid-Atlantic (12.5 ± 6.4) (Figure 2). When considering only the top 25 plastic surgery programs with the greatest medical student publication productivity, the region with the greatest number was the Northeast (8/26, 31%; Figure 2A). There were no significant differences in average medical student first-author publications between regions (Figure 2B). 

Program Rankings

**Figure 3:**
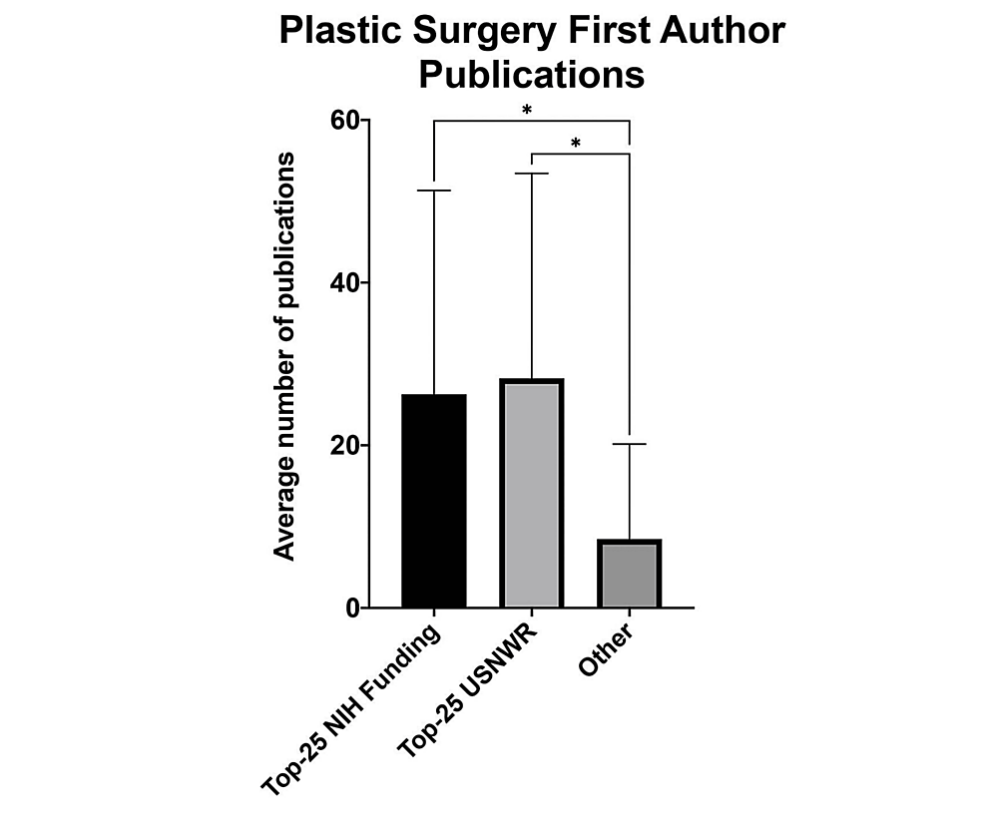
Systematically ranked plastic surgery programs have higher medical student first-author publication volumes. Plastic surgery departments that comprise top 25 programs as measured by NIH funding and USNWR ranking have higher mean publications compared with all other plastic surgery programs. ^*^P < 0.001 by the Student's t-test. NIH, National Institutes of Health; USNWR, U.S. News & World Report

**Figure 4:**
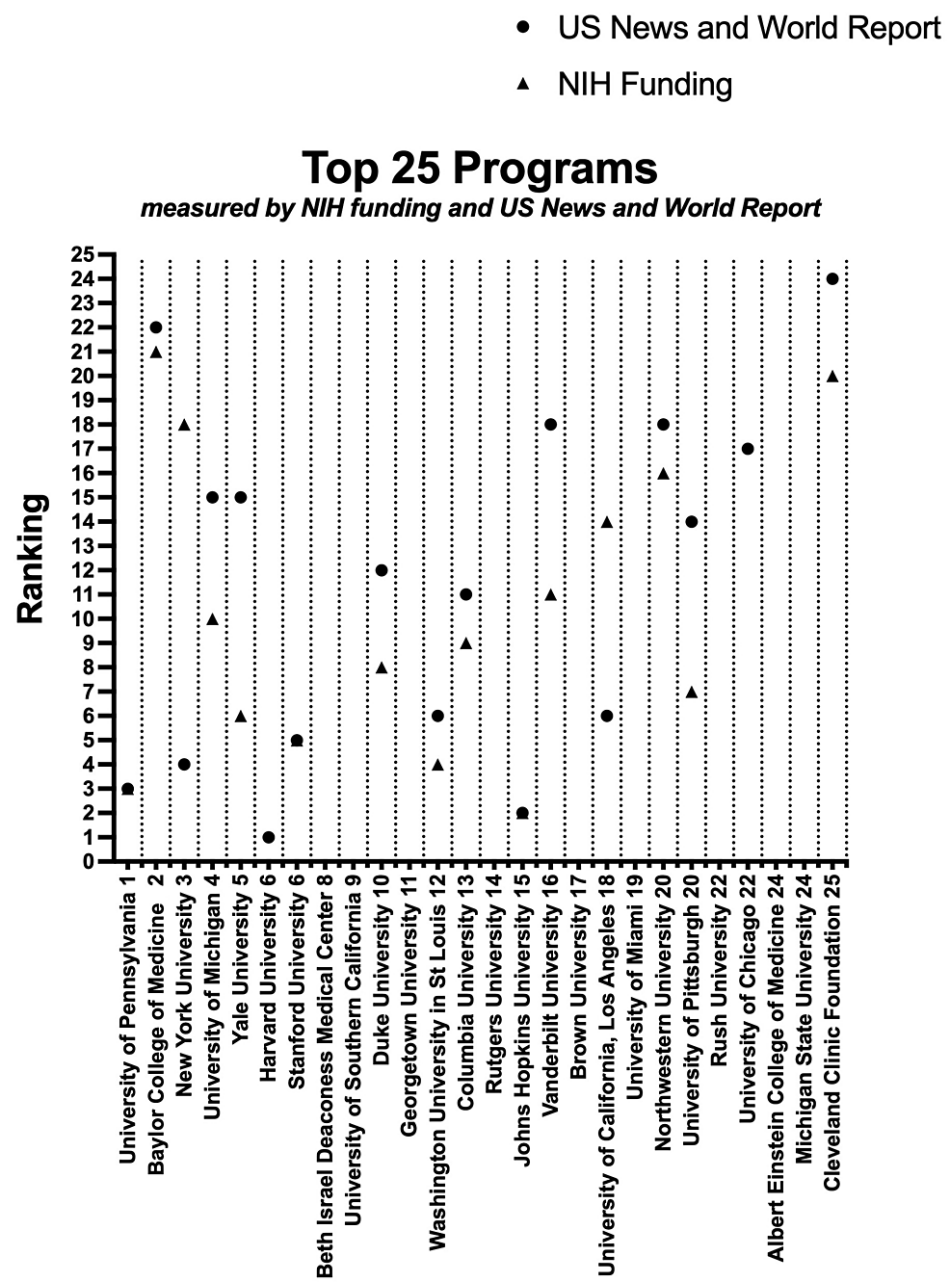
Programs with most academically productive medical students are considered top tier by published ranking systems. Scatterplot representing USNWR ranking and NIH funding for the programs with the greatest number of medical student first-author publications from January 1, 2016, to December 31, 2020 (*n* = 25). USNWR, U.S. News & World Report; NIH, National Institutes of Health

Plastic surgery programs were stratified into groups based on program association with medical schools in the top 25 USNWR Research rankings in 2021 and the top 25 medical school NIH funding dollars acquired (Figure 3). Of the top 25 programs based on medical student first-author publications, 15 (60%) were also ranked in the top 25 by both USNWR ranking and NIH funding. The average (±SD) number of medical student first-author publications at the top 25 USNWR institutions was 26.28 (±25.0). The average (±SD) number of medical student first-author publications at the top 25 NIH-funded institutions was 28.24 (±25.2). The average number of medical student first-author publications outside of the top 25 USNWR and NIH-funded institutions were 8.42 (±11.7) and 7.6 (±10.0), respectively. The differences in medical student first-author publications between the top 25 and the rest of the field were statistically significant (*P* < 0.001) (Figure 4).

Analysis

Research in Context

This study aimed to provide objective data regarding medical student academic productivity in plastic surgery, as well as analyze this academic productivity against various associated factors. We found that 10 integrated plastic surgery programs in the United States are responsible for producing the majority of total medical student first-author publications. The top program was the University of Pennsylvania with 81 medical student first-author publications during the study period. Of the top 25 most productive programs, the Northeast was the most common geographic location (*n *= 8), but there was no significant difference between the average production of medical student first-author publications between regions. We found that there is a positive correlation (*R* = 0.54, *P* < 0.001) between the number of faculty in the department and the number of medical student first-author publications during the study period. We also found that programs ranked highly by USNWR and NIH funding produce more medical student first-author publications than the rest.

As with most of the *competitive* specialties, objective differentiators play a substantial role in the evaluation of applicants. *Demonstrated involvement and interest in research* is currently ranked highly by program directors when determining whom to invite for interviews [7], and with the upcoming changes to the scoring of the USMLE exams, it is not hard to imagine that research productivity will increase in importance to program directors when stratifying applicants [8]. Research production does not just facilitate program director stratification, however. Reading the literature to develop ideas before publishing is a critical skill for learning the fundamental knowledge of the field and approaching possible mentors with research ideas or partially completed manuscripts is a strong strategy for initiating these important relationships. Our analysis of medical student first-author publications in plastic surgery may be a useful resource for current and future medical students who desire a career in plastic surgery and, thus, need to accumulate the requisite research output and mentorship to make them competitive residency applicants and successful academic surgeons.

Despite the high relative importance to future residency program directors, there is a paucity of data regarding pre-residency (i.e., undergraduate education, research *gap* years, and medical school) publication efficacy in plastic surgery. There has been plenty of literature in recent years to gauge the academic productivity of plastic surgery programs by assessing publication rates, industry funding, bibliometric indices, professional reputation, author gender, and academic faculty retention rates of graduates [9-12]. However, these analyses provide data for residents, fellows, and faculty to use in their practices but overlook medical students or combine them with residents. With the ever-increasing competitiveness of the plastic surgery match and changes to board scoring, it is now more important than ever to provide quality research productivity data for medical students to use when setting and striving toward residencies.

Increasingly, authors have recognized the importance of providing medical students with access to data when considering their specialty and residency goals. There have been analyses of medical student academic productivity in dermatology [13,14], neurosurgery [4,6], orthopedic surgery [15], otolaryngology [16], pediatrics [17], and plastic surgery [1]. In relation to our study, Mellia et al. conducted an analysis involving 301 fourth-year medical students entering integrated plastic surgery residencies. They discovered that research productivity, especially in terms of total publications and first-author publications, was linked to an increased likelihood of securing a higher-tier integrated plastic surgery residency [1]. Specifically, the authors found that the average number of publications and *H*-index (a measure of productivity and impact) of their study population (integrated plastic surgery matriculants) were 2.43 and 1.01, respectively. Our current research is a natural extension of this work by providing data to current and future medical students about what programs will best enable them to achieve their professional goals.

These results are not without limitations. Our study is a retrospective literature search, so it is possible that some relevant manuscripts were unintentionally excluded. There were instances where identifying the degree of a manuscript's first author proved challenging, despite making every effort to ascertain the most accurate answer possible. Additionally, some medical students published more than one first author, which could lead to bias. Occasional irregularities were also observed with the listings of plastic surgery faculty on departmental websites as a few institutions did not comprehensively list their plastic surgery faculty on the departmental page. Additionally, in certain departments, dermatologists, otolaryngologists, or podiatrists may also be listed as plastic surgery faculty, though they did not count in our study. In these instances, every effort was maintained to ensure accurate lists of academic plastic surgery faculty. One of the main metrics used to compare programs involved distinguishing programs that were deemed top-tier programs based on NIH funding and USNWR. Though not perfect, these are two widely recognized sources that objectively rank programs and measure research output, and the use of these two sources in identifying top-tier programs that are research-heavy prevents heterogeneity. Finally, from a holistic perspective, there are many other ways to assess medical student research contributions and plastic surgery aptitude than just first-author publications, such as the quality of the published article, the role of the author in the team, the journal's rank and impact factor, the author's *H*-index, total citation number, acceptance into honor societies, distinctions for clinical care, and emotional intelligence. Nevertheless, this is the first study to our knowledge that provides objective conclusions on the programs producing the greatest number of medical student first-author publications and should be a reference to medical students as they strategize their successful match into plastic surgery.

Statistical Analysis

Descriptive statistics were reported as a mean and SD for continuous variables or frequency and percentage for categorical variables. Comparisons of means were performed with a two-tailed t-test; comparisons of proportions were performed with an N-1 chi-square test. Groups were compared by one-way analysis of variance (ANOVA) combined with Tukey post hoc analysis. Categorical data were correlated with the Pearson correlation coefficient, when appropriate. Before performing any tests, statistical significance was defined with a *P*-value < 0.05. All statistical analyses were performed using commercially available software, including JMP 13 (SAS Institute, Cary, NC, USA). Figures were created with Prism 7.0a (GraphPad Software, San Diego, CA, USA).

## Conclusions

Many medical student first-author publications in the United States are produced by 10 plastic surgery programs, and programs associated with higher-ranking medical schools produce greater numbers of medical student first-author publications. A substantial proportion of these plastic surgery programs are located in the Northeast region of the United States. Our analyses of medical student first-author publications in plastic surgery should serve as a highly useful guide to accumulating meaningful research experiences for current and future medical students when strategizing a successful match.

## References

[1] Mellia JA, Jou C, Rathi S, Perzia BM, Morel A, Azoury SC, Fischer JP (2021). An in-depth analysis of research output in successful integrated plastic surgery match applicants and factors associated with matching at top-ranked programs. J Surg Educ.

[2] Schultz KP, Shih L, Davis MJ, Reece EM, Buchanan EP, Maricevich RS, Winocour S (2020). Integrated plastic surgery applicant review: important factors and selection criteria. Plast Reconstr Surg Glob Open.

[3] Oleck NC, Gala Z, Weisberger JS, Therattil PJ, Dobitsch AA, Ayyala HS, Lee ES (2020). Relevance of academic productivity in the assessment of integrated plastic surgery applicants. J Surg Educ.

[4] Price G, Lakomkin N, Kamat S, Baron RB, Scherschinski L, Hadjipanayis C (2021). Medical student publications in neurosurgery: at which U.S. academic institutions do medical students publish most?. World Neurosurg.

[5] Chitti B, Pham A, Marcott S, Wang X, Potters L, Wernicke AG, Parashar B (2018). Temporal changes in esophageal cancer mortality by geographic region: a population-based analysis. Cureus.

[6] Wadhwa H, Shah SS, Shan J (2019). The neurosurgery applicant's "arms race": analysis of medical student publication in the Neurosurgery Residency Match. J Neurosurg.

[7] (2022). Results of the 2020 NRMP Program Director Survey. http://www.nrmp.org/wp-content/uploads/2022/01/2020-PD-Survey.pdf.

[8] Lin LO, Makhoul AT, Hackenberger PN (2020). Implications of pass/fail step 1 scoring: plastic surgery program director and applicant perspective. Plast Reconstr Surg Glob Open.

[9] Ruan QZ, Cohen JB, Baek Y (2018). Does industry funding mean more publications for subspecialty academic plastic surgeons?. J Surg Res.

[10] Davis MJ, Abu-Ghname A, Agrawal N, Reece EM, Winocour SJ (2020). Impact factor, h-index, and alternative metrics: how should we measure the impact of publications in plastic surgery?. Plast Reconstr Surg.

[11] Roy E, Egro FM, Zalewski A, Smith BT, Losee JE, Nguyen VT (2020). Influence of residency training on research productivity and plastic surgery career. Ann Plast Surg.

[12] Bucknor A, Peymani A, Kamali P (2019). International and geographic trends in gender authorship within plastic surgery. Plast Reconstr Surg.

[13] Stratman EJ, Ness RM (2011). Factors associated with successful matching to dermatology residency programs by reapplicants and other applicants who previously graduated from medical school. Arch Dermatol.

[14] Wang JV, Keller M (2016). Pressure to publish for residency applicants in dermatology. Dermatol Online J.

[15] Campbell ST, Gupta R, Avedian RS (2016). The effect of applicant publication volume on the orthopaedic residency match. J Surg Educ.

[16] Kohlert S, Zuccaro L, McLean L, Macdonald K (2017). Does medical school research productivity predict a resident's research productivity during residency?. J Otolaryngol Head Neck Surg.

[17] Gupta R, Norris ML, Barrowman N, Writer H (2017). Pre-residency publication and its association with paediatric residency match outcome-a retrospective analysis of a national database. Perspect Med Educ.

