# Correction: A Review of Medical Student First-Author Publications in Plastic Surgery

**DOI:** 10.7759/cureus.c162

**Published:** 2024-03-01

**Authors:** Abhinav Lamba, Matthew D Rich, Joseph D Quick, Thomas J Sorenson, Ruth J Barta, Warren Schubert

**Affiliations:** 1 Department of Surgery, University of Minnesota Medical School, Minneapolis, USA; 2 Department of Plastic Surgery, New York University (NYU) Grossman School of Medicine, New York, USA; 3 Department of Craniofacial and Plastic Surgery, Gillette Children’s, Saint Paul, USA; 4 Department of Plastic and Hand Surgery, Regions Hospital, Saint Paul, USA

This article has been corrected to change three mentions of "Baylor University" to "Baylor College of Medicine".

